# Simulation and Prediction of Springback in Sheet Metal Bending Process Based on Embedded Control System

**DOI:** 10.3390/s24237863

**Published:** 2024-12-09

**Authors:** Jinhan Xu, Jun Yan, Yan Huang, Dawei Ding

**Affiliations:** 1 College of Automation & College of Artificial Intelligence, Nanjing University of Posts and Telecommunications, Nanjing 210023, China; b22051027@njupt.edu.cn (J.X.); yanjun_990521@163.com (J.Y.); 2 Jiangsu Yawei Machine Tool Co., Ltd., Yangzhou 225200, China; huang.yan@yawei.cc

**Keywords:** bending machine, control system, springback, prediction model, error compensation

## Abstract

Amidst the accelerating pace of automation in sheet metal bending, the need for small-batch, multi-varietal, efficient, and adaptable production modalities has become increasingly pronounced. To address this need and to enhance the efficacy of the bending process, this study presents the design and development of an embedded soft PLC (Programmable Logic Controller) rooted in the Codesys development platform and leveraging the ARM Cortex-A55 architecture. This controller employs the EtherCAT communication protocol to facilitate seamless and efficient interactions with fully electric servo-driven CNC (Computerized Numerical Control) bending machinery. To mitigate the challenge of bending springback errors, a finite element simulation model is constructed and refined through the application of ALE (Arbitrary Lagrangian-Eulerian) adaptive grid technology, thereby bolstering simulation precision. Subsequently, an enhanced WOA-BP (Whale Optimization Algorithm—Backpropagation) model, integrating Latin hypercube sampling and neural network techniques, is deployed to anticipate and counteract these springback errors. Experimental outcomes demonstrate that the proposed methodology effectively constrains the final forming angle deviation to within 0.3°, significantly enhancing the reliability and precision of the bending system. This achievement not only underscores the technical feasibility but also contributes to advancing the frontier of sheet metal bending automation.

## 1. Introduction

Sheet metal bending, a pivotal process in cold metalworking, holds paramount significance in industries such as home appliances and automotive manufacturing due to its strengths in high durability, lightweight construction, cost-effectiveness, and ease of manipulation [1,2,3]. As a vital industrial tool for this purpose, the bending machine has garnered widespread adoption in sheet metal processing due to its straightforward operation and robust process adaptability [4,5,6]. In the digital age, where consumer preferences diversify rapidly, the market’s appetite for personalized sheet metal components intensifies daily, necessitating the bending machinery’s compatibility with production paradigms characterized by small batch sizes, diverse product lines, and swift mold alterations [7,8]. However, conventional bending control systems, predominantly reliant on generic PLC-based numerical controls, exhibit limitations in terms of elevated costs, limited flexibility, and challenges in aligning with contemporary production demands. These drawbacks underscore the need for advancements in bending machine control systems to better cater to the evolving landscape of manufacturing.

Embedded software PLC technology introduces novel avenues for the intelligent management of bending machines while allowing for the leverage of its strengths, including swift real-time responsiveness, customizable software and hardware components, streamlined programming interfaces, seamless network integration, robust compatibility, and cross-platform operational capabilities [9]. Researchers worldwide have made strides in exploring and implementing embedded soft PLC control systems. For example, Zhao et al. [10] devised an embedded soft PLC system that effectively achieved the real-time control of die-casting machinery. Additionally, Xu et al. [11], through their work on an omnidirectional mobile robot control system based on the Codesys platform, adeptly addressed stability challenges in robotic motion. Furthermore, the tile-laying robot control system developed by Du et al. [12] exemplifies the promising application of EtherCAT communication technology in automating equipment. However, a notable limitation of these studies is their focus on specific industrial equipment, which may diverge from the practical requirements of bending machines, thereby hindering direct applicability to their control.

Sheet metal bending processes often encounter the pervasive challenge of springback, a phenomenon wherein the material partially reverts to its original configuration post-bending due to the relief of internal stresses [13]. This springback effect inherently results in a final molding angle that exceeds the anticipated or desired outcome, posing a challenge to achieving precise dimensional accuracy in practical manufacturing scenarios. Consequently, the accurate prediction and compensation for springback are crucial factors influencing the quality of sheet metal bending products.

To mitigate the rebound phenomenon and achieve the desired bending angle, various strategies are commonly employed, including the adjustment of the bending angle, test bending procedures, the augmentation of bending forces, and the extension of holding periods [14]. Chen et al. [15] conducted a comprehensive study investigating the correlation between the servo bending machine’s motion profile and sheet metal springback, subsequently proposing an optimized motion curve. Using DEFORM software simulations, their experimental findings validated the efficacy of this motion curve in significantly reducing springback. Yang et al. [16] theoretically investigated the influence of material attributes, processing techniques, and tooling on sheet metal springback, offering recommendations for its reduction. Trieu et al. [17] leveraged ABAQUS simulations to develop a predictive model for SS400 steel plate V-bend springback, subsequently confirming its accuracy through empirical validation. Similarly, Sharm et al. [18] examined the impact of the upper die fillet radius on springback, discovering that a smaller radius effectively diminished the phenomenon. Ouled et al. [19] employed numerical simulations to explore the interplay between springback and key parameters, such as the upper die fillet radius, lower die opening width, and sheet thickness. Trzepieciński et al. [20] utilized a GA-ANN model to forecast steel plate springback angles, with experiments revealing that the extent of upper die pressing was the primary influencing factor. Buang et al. [21], focusing on the V-die bending process, highlighted the significance of punch travel and die gap on springback behavior, with punch travel yielding the most statistically significant effect (*p* = 0.003). Collectively, these studies underscore the multifaceted nature of sheet metal springback, which is intricately linked to both bending process parameters and inherent material properties.

Domestic scholars have intensified their efforts in investigating the intricacies of springback through the application of simulation methodologies. Zhou et al. [22] constructed a comprehensive training sample dataset by leveraging finite element simulations and subsequently employed a BP neural network to predict sheet metal springback. Their findings underscored the profound influence of uncontrollable process variables on the springback phenomenon. Zhao et al. [23] introduced a novel real-time bending angle control strategy rooted in force feedback principles, which was validated through ABAQUS simulation experiments and a recurrent neural network prediction model, offering a robust solution for precision bending. Yu et al. [24] advanced a data-driven approach for modeling punch stroke corrections in trial bending, demonstrating the superiority of neural network models over traditional dimensional analysis models in terms of accuracy and production efficiency enhancements. Furthermore, Fu et al. [25] formulated a Grey Prediction GM (1,1) model to predict the upper die pressing force and resulting bending angle in free bending scenarios. The model’s predictive accuracy was exemplary, with a maximum error of merely 0.14%, as verified within ABAQUS simulations.

In summary, extensive research employing diverse methodologies, both domestically and internationally, has been conducted to analyze the multifaceted impact of various factors on springback, leading to the development of a predictive model. Nevertheless, the intricacies of sheet metal springback encompass numerous variables, rendering a complete simulation of the process through limited experiments inherently challenging. Furthermore, employing real-world bending data as the basis for predictive model training datasets incurs significant costs and hampers the feasibility of rigorous variable control, thereby limiting the comprehensiveness and accuracy of such models.

This study presents the design of an integrated intelligent control system for a bending machine featuring springback prediction functionality. This research focuses on an all-electric servo bending machine, custom-designed in our laboratory, serving as the platform. At the heart of this control system lies a Cortex-A55 chip, complemented by an embedded soft PLC controller. Leveraging finite element simulation and advanced intelligent algorithms, the system predicts bending springback with precision, thereby mitigating forming errors effectively. Experimental validation through bending tests underscores the system’s efficacy in enhancing bending accuracy and optimizing production processes. This work introduces a novel approach to the intelligent control of bending machines, offering a practical solution for the industry to improve product quality and efficiency.

## 2. Design of CNC Bending System

### 2.1. Structure of Bending Machines

**Figure 1:**
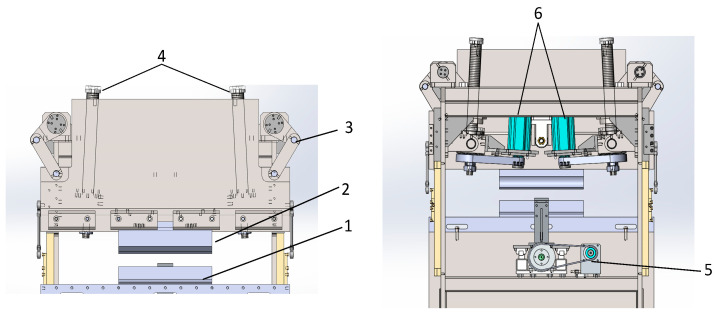
Diagram of the bending machine and component description; 1—lower mold; 2—upper mold; 3—connecting rod structure; 4—rollerscrews; 5—backstop motor; 6—slider motor.

This study employs a self-designed, all-electric servo bending machine as the primary research apparatus. This bending machine incorporates a linkage mechanism, which significantly enhances its bending force by approximately threefold compared with that of conventional bending machinery. Figure 1 illustrates the constructed bending machine, highlighting its innovative design and enhanced capabilities.

**Figure 2:**
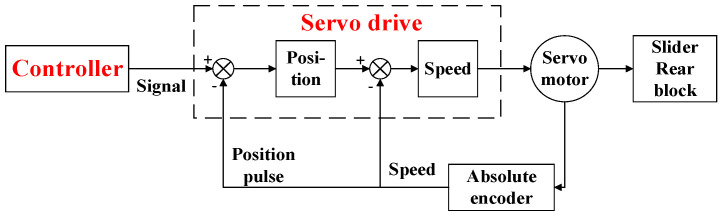
Feedback control of the servomotor.

The slider and backstop of the bending machine operate through linear motion, with the slider employing a dual servo motor system that synchronously drives a roller screw mechanism, whereas the backstop utilizes a direct servo motor drive approach. This configuration, in contrast to the conventional manual plate bending machines reliant on manual positioning, offers enhanced precision and expedited efficiency through servo motor control. The servo motor control program is illustrated in Figure 2, underscoring the technological advancement in automation and precision achieved in this system.

The servo motor operates in conjunction with the encoder’s tail, providing real-time feedback on motor displacement and angular velocity to the servo drive. This feedback signal enables the servo drive to adjust and correct accordingly, thereby realizing a comprehensive feedback control mechanism within the entire control system. This streamlined approach ensures precision and responsiveness in the servo motor’s operation.

### 2.2. Hardware of Numerical Control System

**Figure 3:**
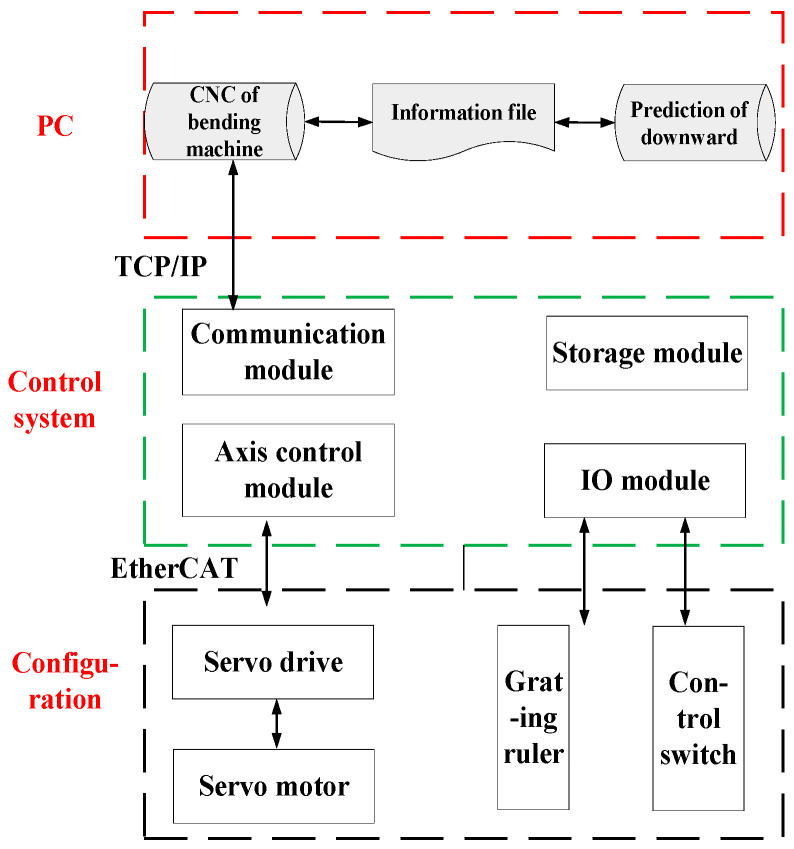
Hardware framework of the bending machine control system.

In the proposed design framework, the dedicated CNC system tailored for bending machines serves as the cornerstone, providing essential hardware resources and a robust data transmission interface. This hardware layer functions predominantly as a conduit for seamless data communication and conversion between the CNC processing zone of the bending machine and its structural system. The core components comprising this layer encompass the controller, an EtherCAT bus communication submodule, servo drivers, and servo motors, among others. The architecture of this system’s hardware configuration is illustrated in Figure 3, which provides a visual representation of the key elements and their interconnections.

The upper computer serves a dual purpose, primarily focusing on two core functionalities: firstly, the development of the NC system, leveraging the robust Codesys software platform, and, secondly, the anticipation of the pressing force applied by the upper die in the bending machine, achieved through an enhanced WOA-BP neural network model. This predictive system and the NC system engage in seamless data exchange by means of file sharing, ensuring efficient communication and the integration of functionalities.

The hardware architecture of the bending machine’s control system comprises a controller and an IO communication expansion module, both essential components that enable EtherCAT and IO communication functionalities. At the core lies the ARM-based embedded controller, specifically designed and implemented in this study, while the IO communication module integrates Bev’s EK1110 and its corresponding expansion module. The controller assumes the primary role of regulating the servo motor’s movements by transmitting precise control signals to the servo driver. Additionally, it incorporates a grating ruler for feedback purposes, enabling real-time adjustment and maintaining the slider’s exact position on the bending machine, thereby enhancing operational accuracy and precision.

The controller hardware employs an OK3568 series standard development board featuring an RK3568 processor. This processor boasts a powerful four-core 64-bit Cortex-A55 architecture, complemented by an abundance of onboard peripheral resources and interfaces. For versatility, it supports both RS232 protocol and Ethernet, facilitating seamless program flashing and data exchange. Table 1 provides a comprehensive overview of the key parameters of the OK3568 development board.

The I/O communication module utilizes Bev’s EK1110 unit to extend the EtherCAT network, incorporating digital input (EL1809), digital output (EL2809), and grating ruler control (EL5001) modules in a streamlined linear topology configuration, as detailed in Table 2. This arrangement ensures efficient data communication and control capabilities.

### 2.3. Bending Machine Controller Design

**Figure 4:**
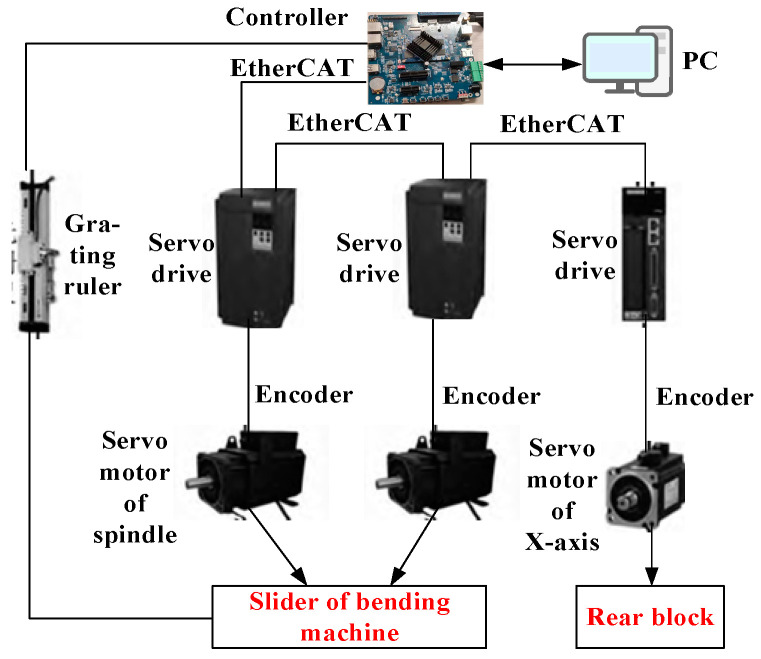
Structure of embedded soft PLC control system.

The embedded control platform comprises an RTS (Real-Time System)-based soft PLC system, wherein the master and slave stations communicate seamlessly via the EtherCAT bus protocol, ensuring efficient data transfer and control synchronization. The EtherCAT master station, developed atop an ARM development board, primarily orchestrates motion control program execution, hardware integration, and system resource allocation. Meanwhile, the EtherCAT slave station comprises three sets of Huichuan IS620N servo drivers and their corresponding servo motors, interconnected in a linear topology configuration on the bus. The architecture of this embedded soft PLC control system is illustrated in Figure 4.

In this study, the Feiling OK3568 development board serves as the primary hardware platform for the development of the EtherCAT master station, with the operating system directly leveraging the Feiling Forlinx Desktop OS. Following the successful burning of the system image onto the development board, the process of constructing and configuring the system environment commences as follows:

(1) Network card configuration is crucial. The development board utilized in this study features two network ports: Port 0 is designated for communication with the PC, whereas Port 1 serves as the EtherCAT communication interface, facilitating seamless data exchange between the master and slave stations. It is imperative that the IP addresses of these ports are configured within the same network segment.
(2) To support installation, debugging, and production phases, essential software packages, including vim, network tools, and cyclist, must be installed.
(3) To enhance system performance and expedite startup time, a streamlining process is undertaken, involving the removal of redundant packages, disabling of unnecessary services, and optimization of system settings.

Currently, RT Linux is predominantly employed to augment the real-time capabilities of a system. This involves integrating a real-time patch onto the Linux kernel, transforming it into a real-time operating system. The migration process for applying this real-time patch is outlined below:

(1) The development board employed in this study incorporates two network ports: Port 0 facilitates communication with the PC, whereas Port 1 serves as the EtherCAT communication interface, enabling seamless data exchange between the master and slave stations. It is crucial that the IP addresses of these ports are aligned within the same network segment. Before proceeding, the real-time patch file must be copied into the system kernel directory of the development board, specifically within the /OK3568-Linux-source/kernel folder.
(2) Subsequently, the patch command is executed to incorporate the two patch files into the system kernel.
(3) Following this, the kernel source code is recompiled, and the kernel image file is updated to reflect the changes, thereby completing the integration of the real-time patch.

The Hackbench tool is utilized to simulate various load types, while cyclictest software is used to assess the system’s response time to events as they occur. Real-time performance is characterized by the maximum tolerable limits of interruption and scheduling delays.

**Figure 5:**
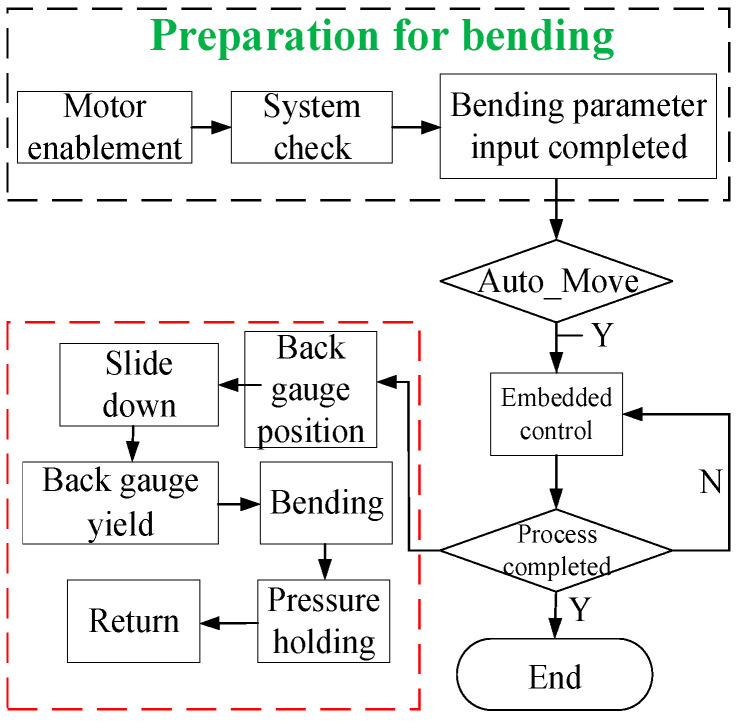
A complete control diagram of embedded system for bending process.

In this context, we primarily focus on comparing the MAX parameters, as the system’s real-time capability is dictated by the peak delay time. By scrutinizing the test outcomes before and after the application of the real-time patch, it is evident that the maximum delay is significantly reduced from 213 μs to 76 μs. This substantial decrease underscores the enhancement of the real-time system, effectively meeting the stringent performance requirements. Compared with the traditional CNC bending system, this one, developed based on soft PLC, has the advantages of low costs and good integration. Additionally, the motion controllers developed based on ARM boards have abundant onboard resources and can integrate machine learning, computer vision, and other functions, with good potential for future development. Then, to achieve complete control of the developed embedded system, a diagram comprising the bending process and embedded control is presented in Figure 5.

## 3. Finite Element Modeling and Simulation of Bending Process

### 3.1. Bending Principle Analysis

**Figure 6:**
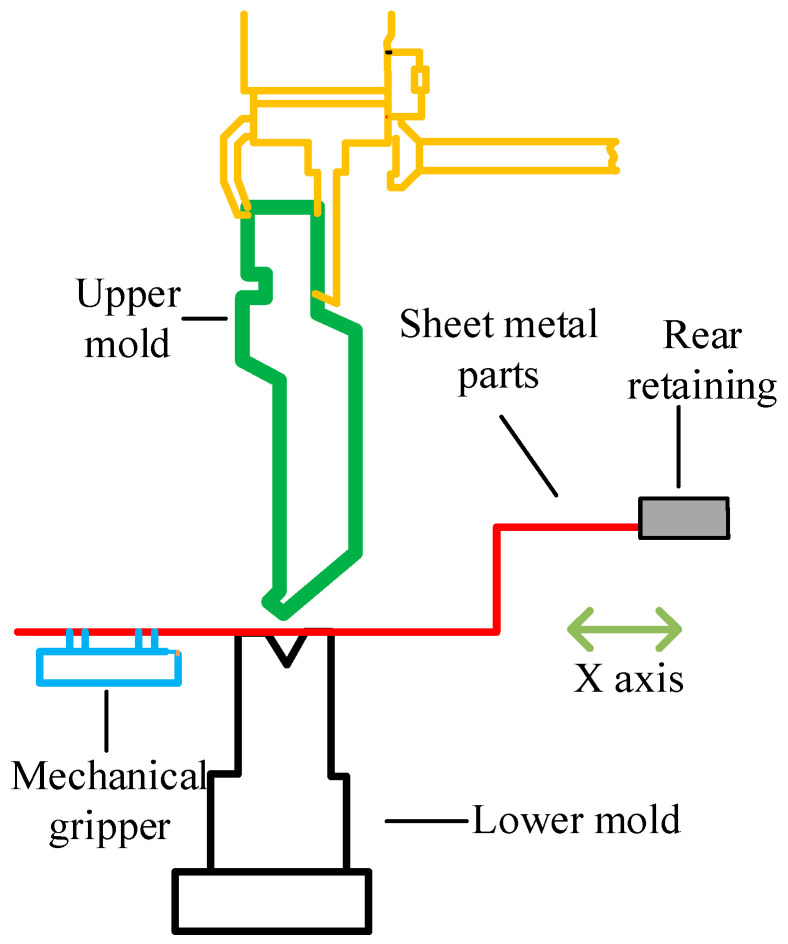
Schematic of traditional free bending process.

The utilization of free bending in sheet metal processing, which achieves diverse bending angles with minimal mold requirements, offers a straightforward and efficient approach, necessitating reduced bending forces. Consequently, free bending is predominantly employed in practical manufacturing scenarios, as exemplified in Figure 6.

### 3.2. Finite Element Modeling

**Figure 7:**
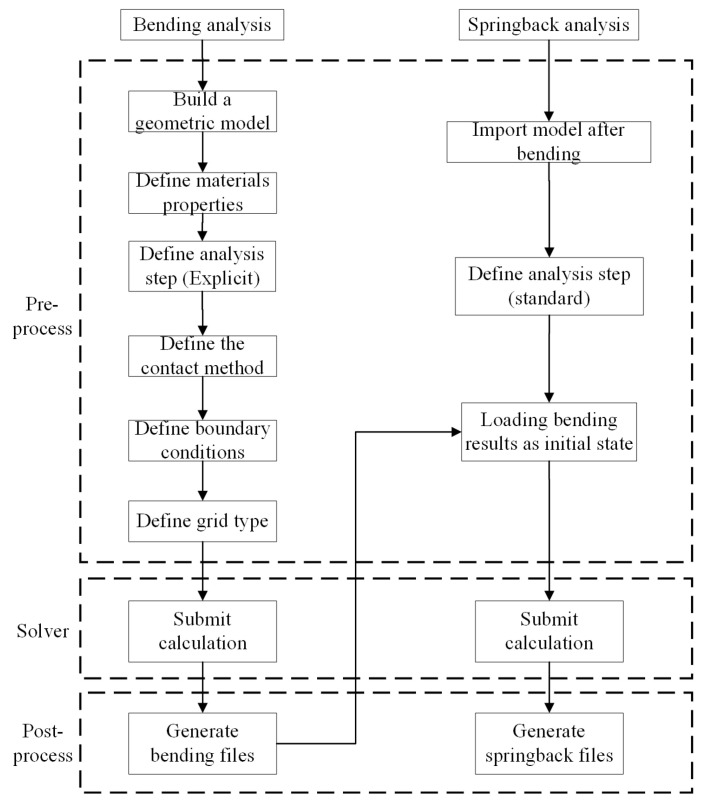
Flowchart of finite element simulation of bending process.

The springback phenomenon in the V-shaped free bending of sheet metal constitutes an intricate, multivariate, and nonlinear dynamic process. Figure 7 presents the flowchart outlining the ABAQUS simulation process for a springback analysis, utilizing an explicit-implicit sequential analysis method to ensure accuracy and efficiency.

**Figure 8:**
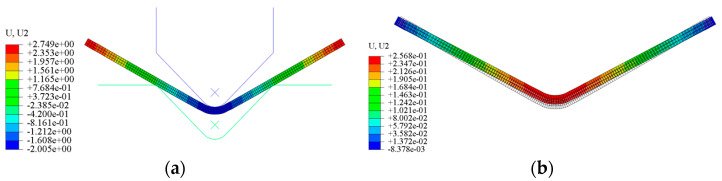
Simulation of the upper mold: (**a**) before unloading; (**b**) after unloading.

The finite element simulation model for V-type free bending employs shell elements as the element type. For the contact interaction between the bending machine’s upper and lower dies and the plate, the penalty contact mode is adopted, with the friction coefficient set to 0.1. The upper and lower dies are designated as the primary contact surfaces, while the plate serves as the secondary contact surface. To mitigate deformation during extrusion, which could compromise model accuracy, the bending machine’s dies are modeled as rigid bodies. The boundary conditions specify that the lower die remains stationary while the upper die descends with a predetermined downward to deform the plate. The mesh is constructed using four-node curved shell elements. The material used in this simulation is stainless steel with an elastic modulus of 180 GPa. Additionally, its thickness is set to 0.5 mm. The loads of downward amount are 2.0 mm, 2.2 mm, 2.4 mm, 2.6 mm, 2.8 mm, and 3.0 mm. The boundary condition is set to be fixed for the lower mold, and the upper mold is moved downward with a fixed amount to cause deformation of the sheet metal. The die parameters correspond to those of a small bending machine, and the plates are modeled as 1 mm thick stainless steel sheets. The simulation outcomes are presented in Figure 8. The legend of U, U2 represents the deformation of sheet metal parts in the simulation process, with the unit of mm.

### 3.3. Analysis of the Effect of Different Meshing Methods

**Figure 9:**
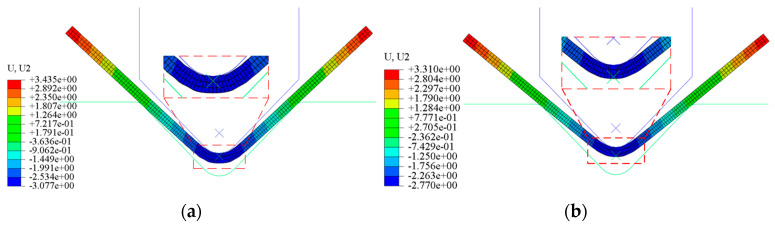
Simulation using different grids: (**a**) not introducing ALE; (**b**) introducing ALE.

ALE methodology represents a widely employed adaptive mesh generation technique that seamlessly blends Lagrangian and Eulerian coordinate frameworks to enhance flexibility in tackling intricate physical phenomena. Given the extensive deformation inherent in bending forming processes, the traditional finite element analysis often encounters mesh distortion and non-convergence issues, jeopardizing the accuracy of the results. To mitigate these challenges, the integration of ALE adaptive mesh generation technology is pivotal, as it dynamically refines the mesh in each subsequent computational step, thereby safeguarding the convergence and precision of the finite element simulation. Figure 9 illustrates the comparative outcomes achieved with the application of ALE adaptive mesh generation.

Before the implementation of ALE adaptive meshing, pronounced mesh distortion was observed in proximity to the contact interface between the upper die’s fillet and the sheet metal component, particularly under conditions of significant sheet deformation. Following the integration of ALE adaptive meshing, however, the mesh density at the bend point increased significantly, resulting in a marked enhancement in mesh smoothness and the complete elimination of distortion. Consequently, the overall mesh quality was substantially improved.

This work used four-node curved shell elements for partitioning, and a simulation was conducted using five different grids separately, as shown in Table 3. The division of grids 1 and 2 gradually increased outward along the centerline of the board, with a denser grid distribution in the midline and a more dispersed grid distribution at both ends. Grids 3, 4, and 5 were evenly distributed. Subsequently, simulations were conducted using each of the distinct meshing methodologies in order to assess their performance.

**Figure 10:**
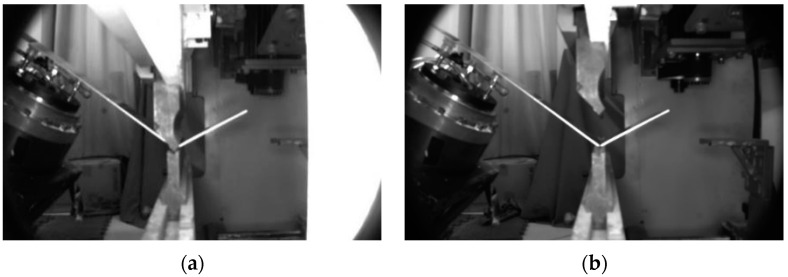
Images of sheet metal parts captured using a Basler camera: (**a**) before unloading; (**b**) after unloading.

The bending process was experimentally captured using a Basler camera, with a focus on capturing the deformation dynamics during the unloading phase of the bending machine’s upper mold. Specifically, two frames, one immediately before and another after the unloading, were selected for precise angular measurements. These measurements, encompassing the pre-unloading and post-unloading angles, are presented in Figure 10, offering a quantitative assessment of the bending process.

**Figure 11:**
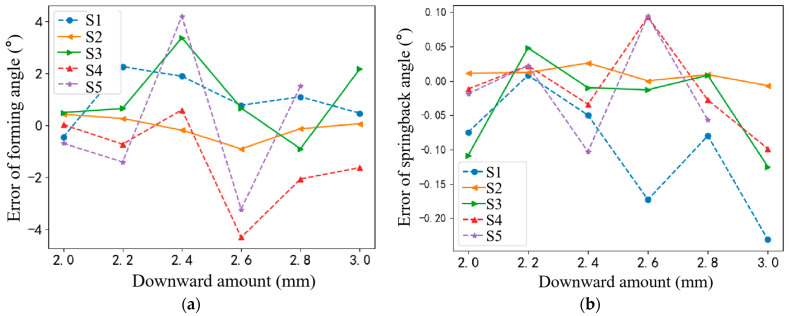
Comparative results of different simulations of (**a**) forming angle; (**b**) springback angle.

A comparative analysis was conducted between the experimentally derived bending angles and five distinct sets of simulation data, each characterized by various grid resolutions. The resulting molding angle deviations and rebound angle inaccuracies are concisely presented in Figure 11, providing a clear visual representation of the comparative performance.

As evident in Figure 11, S2 stands out among the five simulation sets, exhibiting a close correspondence with the experimental outcomes. Specifically, the maximum molding angle error for S2 is a minimal 0.905°, while the rebound angle error remains negligible, at 0.026°. Consequently, the meshing approach employed in S2 is deemed optimal for subsequent experimental endeavors. This validates the accuracy of the finite element model, as S2’s results approximate real-world values with minimal deviations. Thus, the simulation data generated from this model can confidently serve as a surrogate for actual bending data in subsequent analyses and operations.

### 3.4. Analysis of Factors Affecting Sheet Metal Bending Springback

The V-shaped free bending of sheet metal relies on the cooperative action between the upper and lower dies, whereby the bending and springback angles are significantly influenced by the die design and its material properties. This study delves into the primary factors that govern sheet metal springback during bending, encompassing both inherent sheet properties (elastic modulus and thickness) and machine-die characteristics (upper die fillet radius, lower die groove width, and the applied pressure on the upper die). The subsequent figures illustrate the experimental outcomes pertaining to these various influencing factors.

**Figure 12:**
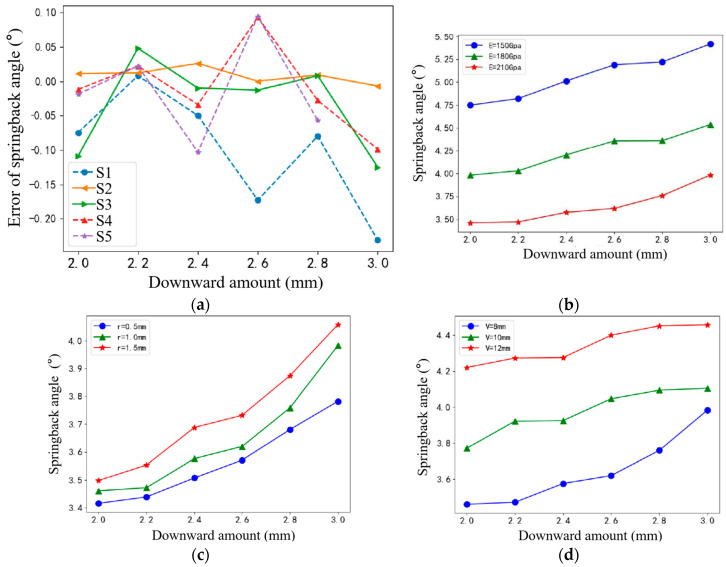
Effect of multiple factors on the springback angle: (**a**) plate thickness; (**b**) elastic modulus; (**c**) corner radius; (**d**) width of lower groove.

Figure 12 illustrates a trend where the bending springback angle of the sheet metal workpiece increases with an increase in the downward amount applied by the bending machine. When comparing simulations across various sheet thicknesses, it is observed that thicker sheets exhibit smaller springback angles, with the springback diminishing further as the downward amount intensifies. Similarly, simulations with a higher elasticity modulus demonstrate a decrease in the springback angle, indicating a positive correlation. Conversely, an increase in the radius of the upper die leads to augmentation in the springback angle. Additionally, simulations with wider lower slot opening widths also exhibit an upward trend in the springback angle, reiterating the influence of die geometry. The key findings are as follows: springback diminishes with thicker sheets and higher downward amounts, a higher elasticity modulus results in reduced springback, and a larger radius of the upper die and wider lower slot opening widths both contribute to increased springback.

These observations underscore the intricate interplay between material properties, die design, and process parameters in determining the springback behavior of sheet metals during bending.

## 4. Improved WOA-BP Neural Network Prediction

### 4.1. Experimental Sample Acquisition

In this study, we selected five pivotal influencing factors for a thorough analysis: the thickness of sheet metal components, the modulus of elasticity, the corner radius of the upper die, the slot width of the lower die’s opening, and the magnitude of the downward amount applied to the upper die. Drawing upon the practical production parameters of the ubiquitous 304 stainless steel sheet metal bending material and the mold specifications of a small, all-electric servo bending machine in our laboratory, we established a benchmark. Based on this benchmark and the defined scope of investigation, the selected parameter ranges are outlined in Table 4, ensuring a focused and methodical approach to our analysis.

The Latin hypercube experimental design represents an efficient, multidimensional parametric sampling approach tailored for experimental design and parametric sensitivity analysis. This methodology is leveraged to ascertain the influence factors governing the samples. Subsequently, finite element simulations were conducted using the derived sample parameters to obtain experimental sample data, a subset of which is presented in Table 5 for illustrative purposes. This streamlined presentation emphasizes the key information while maintaining academic rigor and clarity.

### 4.2. BP Neural Network

The BP neural network, a prevalent artificial neural network model, is trained utilizing the backpropagation algorithm. Comprising an input layer, one or more hidden layers, and an output layer, it exhibits remarkable capability in simulating intricate nonlinear relationships. To approximate function (1), a prediction model based on the BP neural network is devised within the PyCharm environment.

(1) α=f(d,E,r,V,L)

**Figure 13:**
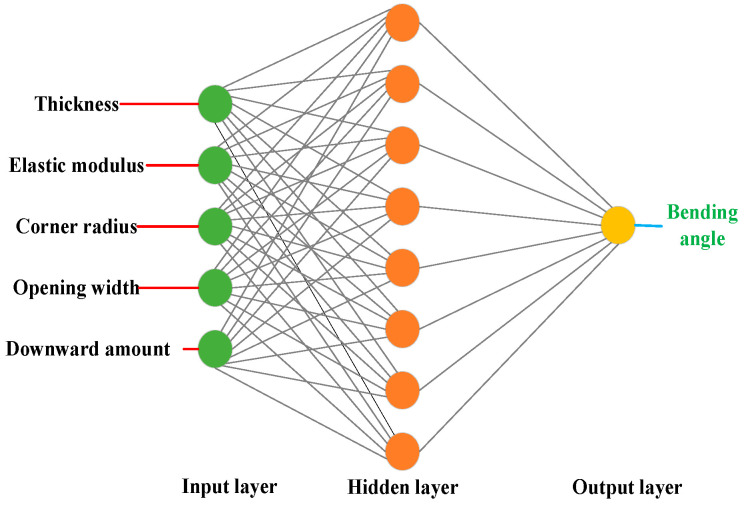
Structure diagram of BP neural network.

The literature suggests that a three-layered BP neural network possesses the capability to accurately fit nonlinear functions to any desired degree of precision. The determination of the optimal number [26] of hidden layer neurons adheres to Formula (2). Consequently, the neural network model depicted in Figure 13 was constructed, adhering to these principles for enhanced clarity and precision.

(2) $$m=\sqrt{n+p}+q$$

where *m*, *n*, and *p* are the number of neurons in the hidden layer, the input layer, and the output layer. Additionally, *q* is an integer between one and ten. Then, m can be determined by combining different choices.

BP network modeling is performed with various numbers of hidden layer nodes, and their corresponding prediction performances are subsequently compared. The outcomes of this comparative analysis are succinctly presented in Table 6, highlighting the key differences and insights. In this neural network model, five inputs of thickness, elastic modulus, corner radius, opening width, and downward amount are considered with only one output of bending angle. Under these circumstances, the amount of data samples are appropriate for training and testing in the industrial applications.

**Figure 14:**
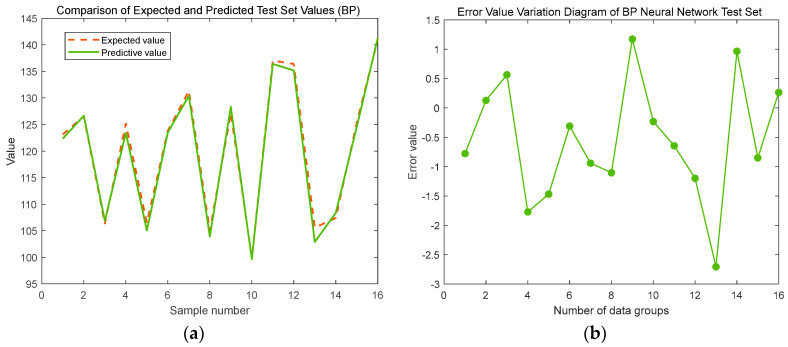
Simulations using BP neural network: (**a**) test and predicted values; (**b**) errors.

As evident in Table 6, an optimal configuration emerges where the hidden layer comprises nine neurons, resulting in the lowest mean square error (MSE) of 0.00060468 for the test set. This configuration signifies the peak performance of the neural network, demonstrating its capability to achieve high prediction accuracy. Consequently, nine neurons in the hidden layer are selected for the subsequent neural network prediction model. The prediction outcomes under this optimal setup are illustrated in Figure 14.

The standard BP neural network model, when evaluated on the test set, exhibits an average error of 0.94374°, a maximum deviation of 2.7058°, and a minimum error of 0.12793° in predicting the bending angle of the V-shaped sheet metal. While the model demonstrates reasonable prediction accuracy, the substantial maximum error precludes it from satisfying the rigorous standards of industrial production.

### 4.3. WOA-BP and Improved WOA-BP

The whale optimization algorithm (WOA) draws inspiration from the hunting tactics employed by humpback whales. Researchers have identified two distinct bubble-net predation methodologies adopted by these whales, labeled as the upward spiral strategy and the double spiral strategy. Notably, the WOA algorithm selectively simulates the upward spiral strategy, replicating the spiral bubble-net predation technique employed by humpback whales in its algorithmic framework. The WOA can be described as follows:

(1) Initialization: The whale optimization algorithm (WOA) initializes the position of the whale population using the following formula, where Xirepresents the location of an individual whale, lb and ub denote the lower and upper bounds of the search space, respectively, and rand is a uniformly distributed random number within the interval [0, 1].
(2) Encircling the Prey Stage: Humpback whales in nature exhibit the ability to locate and encircle their prey. In the context of the WOA, where the optimal position within the search space is unknown, the algorithm assumes that the current best-found candidate position serves as the target prey location. Once this target prey location is identified, the algorithm simulates the behavior of other whales attempting to surround it. The mathematical representation of this encirclement process is formulated as follows:
  (3) $$X\left(t+1\right)=X^{*}\left(t\right)-A\times D$$
  (4) A=2×a×rand−a
  (5) $$D=|C\times X^{*}\left(t\right)-X\left(t\right)|$$
  (6) C=2×rand
  where *t* represents the current iteration count, with *A* and *C* being coefficients. *X* denotes the position of the current solution, whereas *X*^∗^ signifies the position of the current optimal solution. Notably, coefficient *a* undergoes a gradual decrease from 2 to 0 throughout the iterative process.

Due to the randomness in generating the initial weights and thresholds during the training phase of the BP neural network, which inherently influences the structure and performance of the network, the WOA is employed to refine these parameters. The objective is to optimize the initial weights and thresholds, thereby yielding a more stable and efficient WOA-BP neural network model.

The steps involved in optimizing the BP neural network using the WOA are as follows:

Step 1: Initialize the weights and thresholds of the BP neural network.

Step 2: Determine the dimensionality of the decision variables for the WOA and select the mean square error (MSE) as the objective function for optimization.

Step 3: Establish the stopping criteria for the optimization process and utilize the WOA to optimize the weights and threshold parameters of the neural network.

Step 4: Incorporate the optimized weights and threshold parameters into the BP neural network.

Step 5: Train and test the optimized BP neural network and subsequently conduct an error analysis and precision comparison with the non-optimized version to assess its performance improvements.

Despite the WOA demonstrating robust global optimization capabilities and swift convergence in various optimization tasks, its practical applicability is constrained by inherent limitations, necessitating enhancements. To address this, an improved WOA is employed to refine the initial weights and thresholds of the BP neural network. By adopting the training error of the BP network as the individual fitness metric, the optimal initial weights and thresholds are identified. The detailed procedure is outlined below:

Step 1: Initialize the BP neural network by defining its input–output architecture, initial connection weights, and thresholds.

Step 2: Commence the initialization of the enhanced WOA. Translate the initial weight and threshold values from Step 1 into the position vectors of the enhanced WOA. Additionally, set the fundamental algorithm parameters, including the population size (*N*), maximum iteration count (Tmax), initial minimum weight (*w*_1_), initial maximum weight (*w*_2_), and convergence factor (*a*). Notably, the fitness function F(x) for the enhanced WOA is defined as the mean square error between the model’s predicted output and the measured values.

Step 3: Evaluate the fitness of each individual. Identify the position of the optimal fitness value, record its position vector, and designate it as the current optimal individual position (X*).

Step 4: Implement distinct location update strategies contingent upon the value of A. For |A| ≥ 1, apply Formula (3) to update the position for the subsequent generation. Conversely, if |A| < 1, then utilize Formula (4) for the position update.

Step 5: Terminate the optimization algorithm upon reaching the maximum iteration limit or satisfying the specified error accuracy criteria. Subsequently, assign the current optimal parameters to the BP neural network.

### 4.4. Comparison of the Performance of Different Models

The initial population size was set to 30, the maximum iteration count was designated as 50, and the learning rate was determined to be 0.01. Subsequently, a WOA-BP prediction model was established, and efforts were undertaken to further enhance this model.

**Figure 15:**
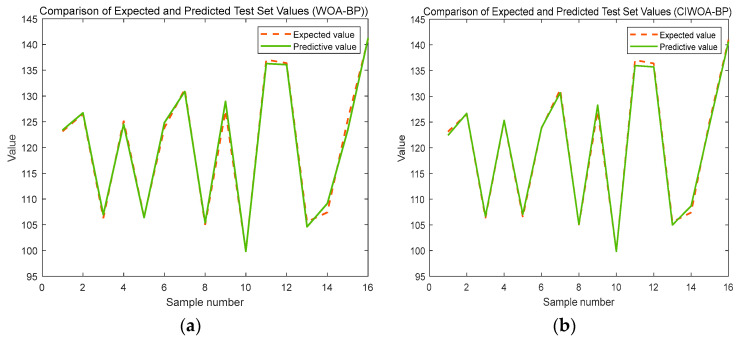
Comparative results using (**a**) WOA-BP algorithm; (**b**) improved WOA-BP algorithm.

The predictive outcomes derived from the base WOA-BP prediction model are presented in Figure 15a, whereas the refined results obtained after improving the WOA-BP prediction model are showcased in Figure 15b.

The mean squared error (MSE), Root Mean Squared Error (RMSE), and Mean Absolute Error (MAE) serve as the primary evaluation metrics to assess the predictive performance of the three distinct prediction models. The outcomes of this evaluation are concisely presented in Table 7, highlighting the performance metrics for each model.

**Figure 16:**
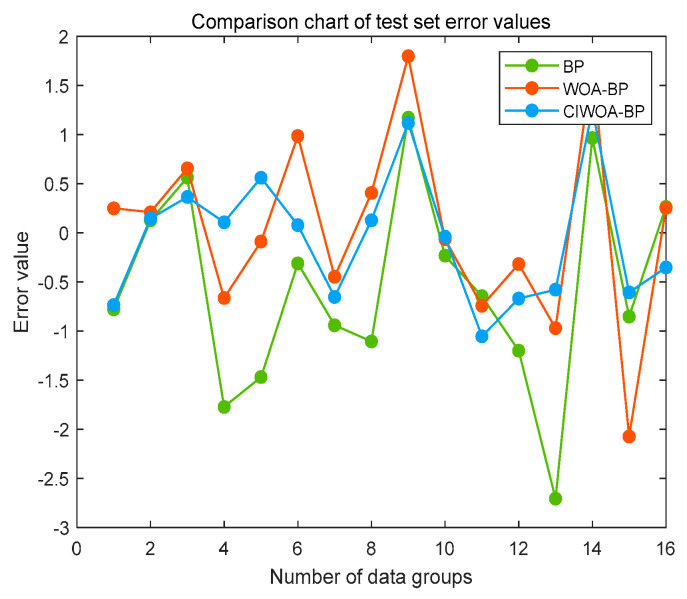
Error comparison of springback angles using different prediction models.

To further validate the predictive performance of the models further, we employed a fixed test set, utilizing consistent random seeds, for data prediction across the three sets of prediction models. The error line graphs, which illustrate the deviations between the predicted and expected values for each of these three models, are displayed in Figure 16.

The error distribution of the enhanced WOA-BP prediction model exhibits a closer proximity to zero, demonstrating superior performance. In comparison with both the standard BP neural network prediction model and the base WOA-BP prediction model, the error curve of the improved WOA-BP model displays a more favorable variation in values, with no significant outliers beyond the data range. Specifically, the maximum error of 1.2537° achieved by the improved model outperforms the 2.073° achieved by the WOA-BP model and the 2.5682° achieved by the BP neural network model. Furthermore, with an average error of 0.52765°, the improved WOA-BP model surpasses the other two in terms of average error, resulting in predictions that are closer to the actual values.

### 4.5. Predictive Model of the Downward Amount in the Upper Mold

During the free bending process of V-shaped sheet metal, the bending machine’s control system regulates the shaping angle by modulating the extent of the upper slider’s downward movement. Consequently, the development of a predictive model to determine the required pressing depth of the bending machine’s upper die is imperative.

Utilizing the data acquired in the preceding section, both a baseline neural network and an enhanced prediction model were devised. The predictive capabilities of these models, each featuring various numbers of hidden layer neurons, were individually scrutinized. The ensuing evaluation outcomes are tabulated in Table 8 for a clear understanding.

As evident in Table 8, the optimal configuration comprises 11 neurons in the hidden layer, resulting in a mean squared error (MSE) of 0.0032989 for the BP neural network prediction model. Leveraging this optimal setup, a BP neural network with a 5-11-1 architecture is established.

**Figure 17:**
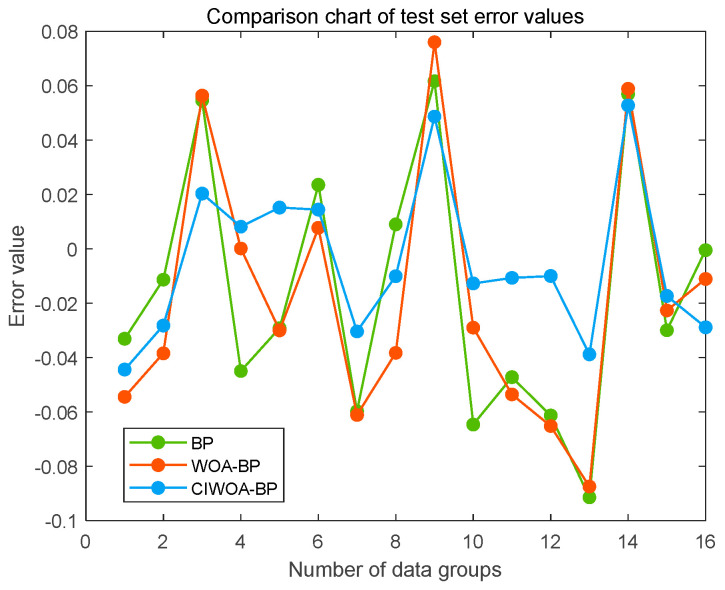
Error comparison of downward amount using different prediction models.

Three distinct prediction models are constructed, each utilizing the basic BP neural network, the WOA-BP neural network, and the improved WOA-BP neural network, respectively, to forecast the downward amount. The predictive accuracy of these three models is then compared, with their prediction errors illustrated in Figure 17.

The improved WOA-BP prediction model exhibits a better prediction performance than both the WOA-BP and the basic BP neural network prediction models, albeit in a nuanced manner. Notably, from an overall perspective, the prediction errors of the improved model are closer to zero, indicating superior prediction effectiveness over the other two. Specifically, the maximum error of 0.052785 mm of the improved WOA-BP model surpasses the 0.087411 mm of the WOA-BP model and the 0.091369 mm of the BP neural network model. Furthermore, the average error of 0.02443 mm achieved by the improved model significantly outperforms the 0.042427 mm achieved by the WOA-BP model and the 0.0431 mm achieved by the BP neural network model, underscoring its enhanced accuracy.

## 5. Experimental Validation

**Figure 18:**
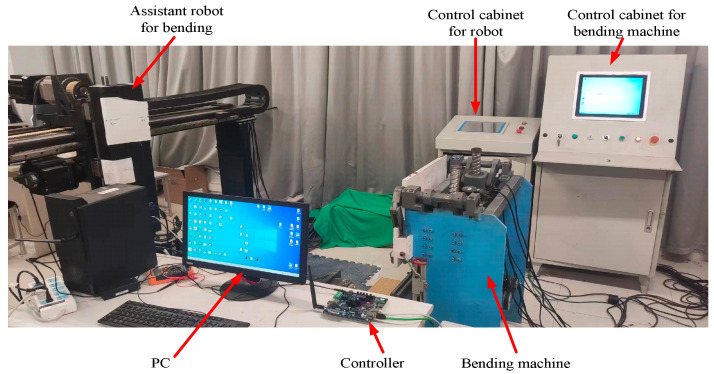
Experimental configuration of the robot-assisted bending system.

To validate the practicality of the bending control system devised in this study, an integrated bending work system was assembled utilizing the existing bending auxiliary robot housed within the laboratory environment. The experimental configuration is depicted in Figure 18.

During the experimental phase, a 1 mm thick stainless steel plate served as the material for actual bending operations. Within the control system’s interactive interface, the bending parameters were precisely configured: a thickness of 1 mm, a length of 60 mm, a setback distance of 30 mm, a pressure holding time of 2 s, and the selection of manual mode for the bending process. Moreover, the controlling and sampling time of this system are both approximately a few milliseconds, thereby scarcely affecting the mechanical motion. Therefore, the developed system is effective in bending the sheet metal parts.

**Figure 19:**
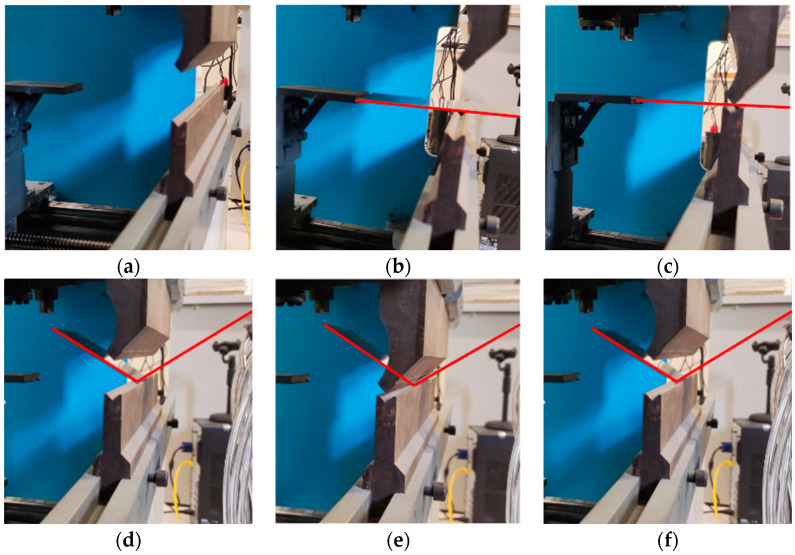
Bending process using the designed system and methods: (**a**) preparation for bending; (**b**) feeding; (**c**) slider down; (**d**) back stopper; (**e**) bending process; (**f**) backhaul.

The operational sequence of the bending machine during the actual processing stage is visually presented in Figure 19.

The experimental outcomes confirm the viability of the system, enabling successful bending of sheet metal components through the implemented bending machine control system. Subsequently, the bending angle was precisely measured using an angle ruler, revealing that the maximum forming angle error of the enhanced WOA-BP neural network model was constrained within 0.3°, indicative of a high prediction accuracy. Furthermore, a traditional process from Ref [27] was adopted with the controlled errors of the bending angle being less than 0.8°. This means that the method proposed in this work can effectively improve the bending accuracy, demonstrating advantages and superiority in the same type of equipment.

## 6. Conclusions

This study presents an embedded soft PLC controller designed specifically for an all-electric servo bending machine laboratory, leveraging the Codesys development environment and ARM Cortex-A55 architecture. The Linux kernel was optimized with real-time patches to enhance system responsiveness. Utilizing ABAQUS simulation software, a V-shaped sheet metal bending model was constructed, addressing mesh distortion issues through the integration of ALE adaptive mesh technology. This refinement improved the bending finite element model and optimized mesh generation accuracy.

Three distinct neural network models, BP, WOA-BP, and an improved WOA-BP, were employed to develop a downward amount prediction model. Notably, the improved WOA-BP model demonstrated superior performance with a maximum error of 0.055371 mm, outperforming both the WOA-BP model (0.087411 mm) and the BP neural network model (0.090791 mm). Experimental validation of sheet metal bending confirmed that this approach effectively controls the angle forming error within 0.3°, significantly enhancing bending stability and reliability. This work provides an effective tool that combines a finite element analysis and neural networks to improve the bending accuracy in industrial engineering. The simulated data and adopted neural networks were not thoroughly recognized, leading to the method’s limitations. In future work, it is necessary to optimize intelligent algorithms to achieve satisfactory computational accuracy using numerous experimental data, thereby enhancing the applicability of this method and promoting the automation procedure in similar manufacturing scenarios.

## Figures and Tables

**Table 1:** OK3568 parameters.

Interface	Description
Display	HDMI, LCD, LVDS
Power supply	DC 12 V
Networking	10/100/1000 Mbps adaptive network port
USB	2-way USB2.0, 1-way USB3.0
Serial port	2-way CAN bus interface, 3-way UART

**Table 2:** I/O parameters.

Extension Module	Function Description
EK1110	Expand EtherCAT network
EL1809	16 channels of digital inputs
EL2809	16 channels of digital outputs
EL5001	Raster scale information acquisition

**Table 3:** Different meshing methods.

Grid 1	Grid 2	Grid 3	Grid 4	Grid 5
0.1–0.2 mm	0.05–0.2 mm	0.1 mm	0.2 mm	0.05 mm

**Table 4:** Influencing factors and their value ranges.

E/GPa	V/mm	L/mm	r/mm	d/mm
150, 180, 210	8, 10, 12	2.0–3.0	0.5, 1.0, 1.5	0.5–1.0

**Table 5:** Data of some experimental samples.

Serial Number	d/mm	E/GPa	r/mm	V/mm	L/mm	α/°
1	0.5	210	1	8	2	126.652
2	0.6	210	1	8	2.1	123.059
3	0.7	210	1	8	3	101.476
4	0.8	210	1	8	2.2	117.911
5	0.9	210	1	8	2.5	110.951
6	1	210	1	8	2.7	102.528
7	0.5	180	1	8	2.2	121.973
8	0.6	210	1	8	2.1	123.059
9	1	210	1	8	3	98.2304
10	0.6	210	1	8	2.6	109.949
11	0.5	210	1	12	2.4	139.838
12	0.6	210	1	8	2	125.764
13	0.5	210	1	10	2.3	132.222
14	0.5	210	1.5	8	2.3	118.637
15	0.5	180	1	8	3	102.977
16	0.5	150	1	8	3	105.118
17	0.5	180	1	8	2.7	108.788
18	0.8	210	1	8	2.7	104.938
19	0.5	210	0.5	8	2.6	114.161
20	0.5	210	0.5	10	2	138.311
…	…	…	…	…	…	…
151	0.6	210	1	8	2.7	107.412

**Table 6:** Mean square error with different numbers of neurons in different hidden layers.

No.	Number of Individuals	Test Set MSE
1	4	0.0012909
2	5	0.0010894
3	6	0.0012803
4	7	0.00089235
5	8	0.00080406
6	9	**0.00060468**
7	10	0.00098316
8	11	0.00063387
9	12	0.0010104

**Table 7:** Comparison of evaluation results of different prediction models.

Model	MSE (%)	RMSE (%)	MAE (%)
BP	0.057	2.384	1.897
WOA-BP	0.028	1.667	1.242
Improved WOA-BP	0.011	1.044	0.775

**Table 8:** Mean square error with different numbers of neurons in different hidden layers.

No.	Number of Individuals	Test Set MSE
1	4	0.0062538
2	5	0.0052425
3	6	0.0055867
4	7	0.0041275
5	8	0.0052358
6	9	0.0058598
7	10	0.0045123
8	11	**0.0032989**
9	12	0.0047449

## Data Availability

Some or all data, models, or codes that support the findings of this study are available from the authors upon reasonable request.
